# Identification of mutations on the *EMD* and *EYA4* genes associated with Emery–Dreifuss muscular dystrophy and deafness: a case report

**DOI:** 10.3389/fneur.2023.1183147

**Published:** 2023-05-12

**Authors:** Ana Karina Zambrano, Elius Paz-Cruz, Santiago Cadena-Ullauri, Patricia Guevara-Ramírez, Viviana A. Ruiz-Pozo, Rafael Tamayo-Trujillo, Rita Ibarra-Castillo, José Luis Laso-Bayas, Nieves Doménech, Adriana Alexandra Ibarra-Rodríguez, Ricardo Hidalgo

**Affiliations:** ^1^Centro de Investigación Genética y Genómica, Facultad de Ciencias de la Salud Eugenio Espejo, Universidad UTE, Quito, Ecuador; ^2^Department of Hemodynamics, Clinical Cardiac Electrophysiologist, Quito, Ecuador; ^3^Instituto de Investigación Biomédica de A Coruña (INIBIC)-CIBERCV, Complexo Hospitalario Universitario de A Coruña (CHUAC), Sergas, Universidad da Coruña (UDC), A Coruña, Spain; ^4^Grupo de Investigación Identificación Genética-IdentiGEN, FCEN, Universidad de Antioquia, Medellin, Colombia; ^5^Centro de Investigación en Salud Pública y Epidemiología clínica (CISPEC), Facultad de Ciencias de la Salud Eugenio Espejo, Universidad UTE, Quito, Ecuador

**Keywords:** genome, muscular dystrophy, deafness, case report, NGS

## Abstract

**Introduction:**

Hearing loss is the most common sensory disability, and it is estimated that 50% of cases are caused by genetic factors. One of the genes associated with deafness is the eyes absent homolog 4 (*EYA4*) gene, a transcription factor related to the development and function of the inner ear. Emery–Dreifuss muscular dystrophy is a rare inherited disease characterized by atrophy and weakness of the humeroperoneal muscles, multi-joint contractures, and cardiac manifestations. It is inherited in an autosomal-dominant, X-linked, or less frequently autosomal recessive manner; one of the genes associated with EDMD is the emerin (*EMD)* gene.

**Case description:**

A total of two Ecuadorian siblings aged 57 (Subject A) and 55 (Subject B) were diagnosed with deafness and an unspecified type of muscular dystrophy based on family history and clinical findings. Next-generation sequencing (NGS) using the TruSight Cardio and Inherited Disease kits at the Centro de Investigación Genética y Genómica CIGG, Universidad UTE, was performed. The genetic analyses showed two mutations: a stop mutation in exon 11/20 (NM_004100.4:c.940G>T) of the *EYA4* gene and a missense mutation in exon 6 (NM_000117.2:c.548C>G) of the *EMD* gene.

**Discussion and conclusion:**

The *in silico* predictions described the *EYA4* variant as likely pathogenic and the *EMD* variant as a variant of uncertain significance (VUS). Moreover, an ancestry analysis was performed using 46 Ancestry Informative Insertion/Deletion Markers (AIM-InDels), and the ancestral composition of subject A was 46% African, 26.1% European, and 27.9% American Indian ancestry, whereas the ancestral composition of subject B was 41.3% African, 38.2% European, and 20.5% American Indian ancestry. The present case report describes two Ecuadorian siblings with a mainly African ancestral component, muscular dystrophy, and deafness phenotypes. Moreover, using next-generation sequencing (NGS), a mutation in the *EMD* and a novel mutation in *EYA4* genes possibly associated with the subjects' phenotype were identified and discussed.

## Introduction

Hearing loss is the most common sensory disability, and it is estimated that 50% of cases are caused by genetic factors (1). As of 2021, more than 200 non-syndromic hearing loss genes have been identified (2). One of the genes associated with deafness is the eyes absent homolog 4 (*EYA4*) gene, a transcription factor related to the development and function of the inner ear (3). The *EYA4* gene is located on chromosome 6q23.2, consists of 21 exons, and encodes a protein of 639 amino acids with two functional domains. The highly conserved eyes absent homologous region (eyaHR) domain composed of 271 amino acids is located at the C-terminal, and the transactivation domain eyes absent variable region (eyaVR), rich in proline, serine, and threonine, is at the N-terminal (1). Moreover, the protein has active and binding sites at residues 375 and 377 (4). Most mutations in the *EYA4* gene are autosomal-dominant (1).

Emery–Dreifuss muscular dystrophy disease (EDMD) is a rare inherited disease, with an incidence of 1–9 in 1,000,000 worldwide (Orphanet Report Series, http://www.orpha.net). EDMD is characterized by atrophy and weakness of the humeroperoneal muscles, multi-joint contractures, and cardiac manifestations (5). EDMD is inherited in an autosomal-dominant, X-linked, or less frequently autosomal recessive manner (5). One of the genes associated with EDMD is the emerin (*EMD*) gene that is located on chromosome Xq28 and is composed of six exons encoding the emerin protein (3). The emerin protein has a *LAP2–emerin–MAN1* (LEM) domain and two regions of interaction with other proteins. The LEM domain is located between residues 1 and 45, the F-actin interaction region between residues 46 and 222, and the catenin–beta 1 (CTNNB1) interaction region between residues 168 and 186 (4, 6). Moreover, the emerin protein participates in biological processes, such as intercellular and intracellular signaling, nuclear structure, chromatin condensation, and epigenetic processes (5). In addition, the emerin protein is involved in cardiac and muscle gene regulation (6).

This case report describes two Ecuadorian siblings with an unspecified type of muscular dystrophy, deafness, and a mainly African ancestral component. Moreover, using next-generation sequencing (NGS), a mutation in the *EMD* gene and a novel mutation in the *EYA4* gene, possibly associated with the subjects' phenotype, were identified.

## Family description

**Figure 1:**
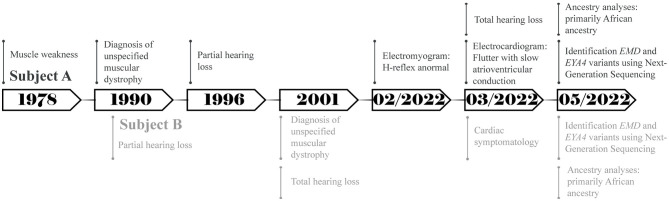
Timeline representing the relevant points of care of both subjects.

**Figure 2:**
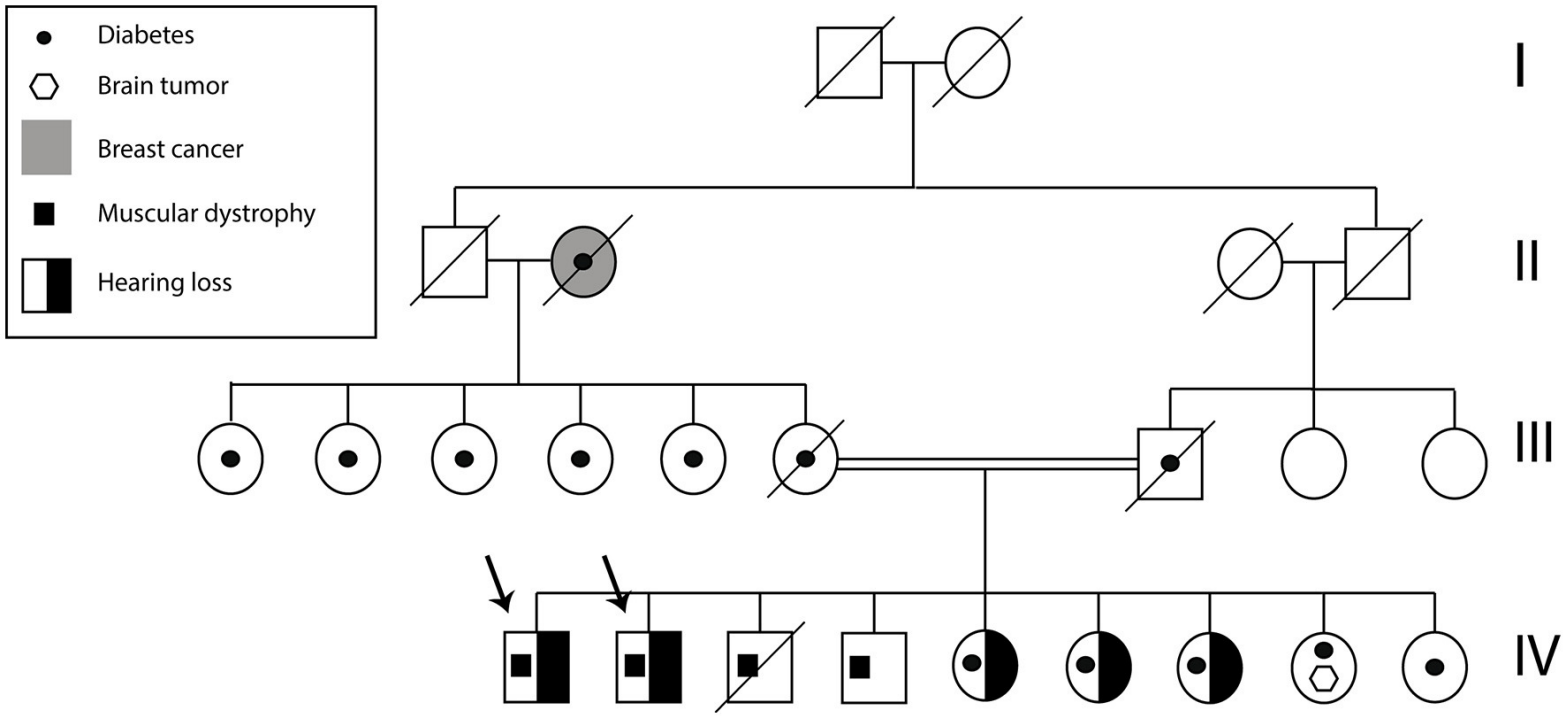
Subject A and B pedigree. The arrows indicate Subject A and Subject B. The family history of health problems is presented in the figure.

A total of two Ecuadorian siblings, aged 57 (Subject A) and 55 years (Subject B), were diagnosed with muscular dystrophy and deafness based on their family history and clinical findings. A timeline describing the relevant data is presented in Figure 1. The family history revealed several diseases, including cancer, hypertension, diabetes, and muscular dystrophy. There are a total of nine siblings born from a consanguineous marriage. Four males were diagnosed with muscular dystrophy, and two suffered hearing loss. Three of the remaining five sisters had moderate hearing loss. Likewise, one of their sisters was infertile and diagnosed with a brain tumor. Moreover, one of the siblings died of heart failure (Figure 2).

### Individual A

Subject A showed muscle weakness since he was 12 and was diagnosed with an unspecified type of muscular dystrophy manifesting joint contractures at about 23. After 7 years, he developed partial symmetrical hearing loss. In February 2022, his electromyogram results showed values outside the normal range (~30 ms) to H-reflex in the two tibial–soleus nerves: the left H-reflex latency was 35.5 and 39.8 ms for the right H-reflex. The chosen treatment method was biperiden *via* the oral route. Additionally, the electrocardiogram showed flutter with slow atrioventricular conduction; amlodipine and angiotensin II receptor blockers were used to alleviate the symptoms. Until May 2022, the subject reported a 30% physical disability and total hearing loss; however, at the last follow-up in January 2023, the symptoms were aggravated and the individual lost his independence.

### Individual B

Subject B showed muscle weakness during adolescence. At the age of 25, he suffered partial symmetrical hearing loss. He was diagnosed with an unspecified type of muscular dystrophy with similar symptoms to Subject A and total hearing loss at 35. The selected treatment method was similar to Individual A, and it consisted of biperiden, angiotensin II receptor blockers, and simvastatin. Additionally, he mentioned cardiac symptomatology and the presence of a heart murmur during the interview. Until May 2022, he had a physical disability of 48%; however, the disability has slowly increased during the last year.

## Results

Genetic testing was performed by next-generation sequencing (NGS) at the Centro de Investigación de Genética y Genómica CIGG, Universidad UTE. Blood samples from the subjects were collected with their informed consent (CEISH-2021-016) in February 2022. The TruSight Cardio Kit was used to sequence 174 genes associated with cardiovascular disease on an Illumina MiSeq with 150-base pair paired-end reads. Genetic analyses showed two mutations: Subjects had a stop mutation in exon 11/20 (NM_004100.4:c.940G>T) and a missense mutation in exon 6 (NM_000117.2:c.548C>G) of *EYA4* and *EMD* genes, respectively. The Variant Interpreter Platform (Illumina) showed that the *EYA4* variant is likely pathogenic, and *EMD* is a variant of uncertain significance (VUS). The genomic analysis results suggested that the type of muscular dystrophy could be EDMD, based on the variant found in the *EMD* gene. Furthermore, a genomic analysis with TruSight Inherited Disease Kit (Illumina) was performed to search for variants in 552 genes associated with inherited disease. The results did not show genetic variants associated with both siblings' clinical phenotypes. These genomic analyses were not performed on the rest of the siblings.

Furthermore, an ancestry analysis was performed using 46 Ancestry Informative Insertion/Deletion Markers (AIM-InDels) according to the Ecuadorian population ethnicity report (7). The ancestral composition of subject A was 46% African, 26.1% European, and 27.9% American Indian ancestry. Subject B has 41.3% African, 38.2% European, and 20.5% American Indian ancestry.

## Discussion

**Figure 3:**
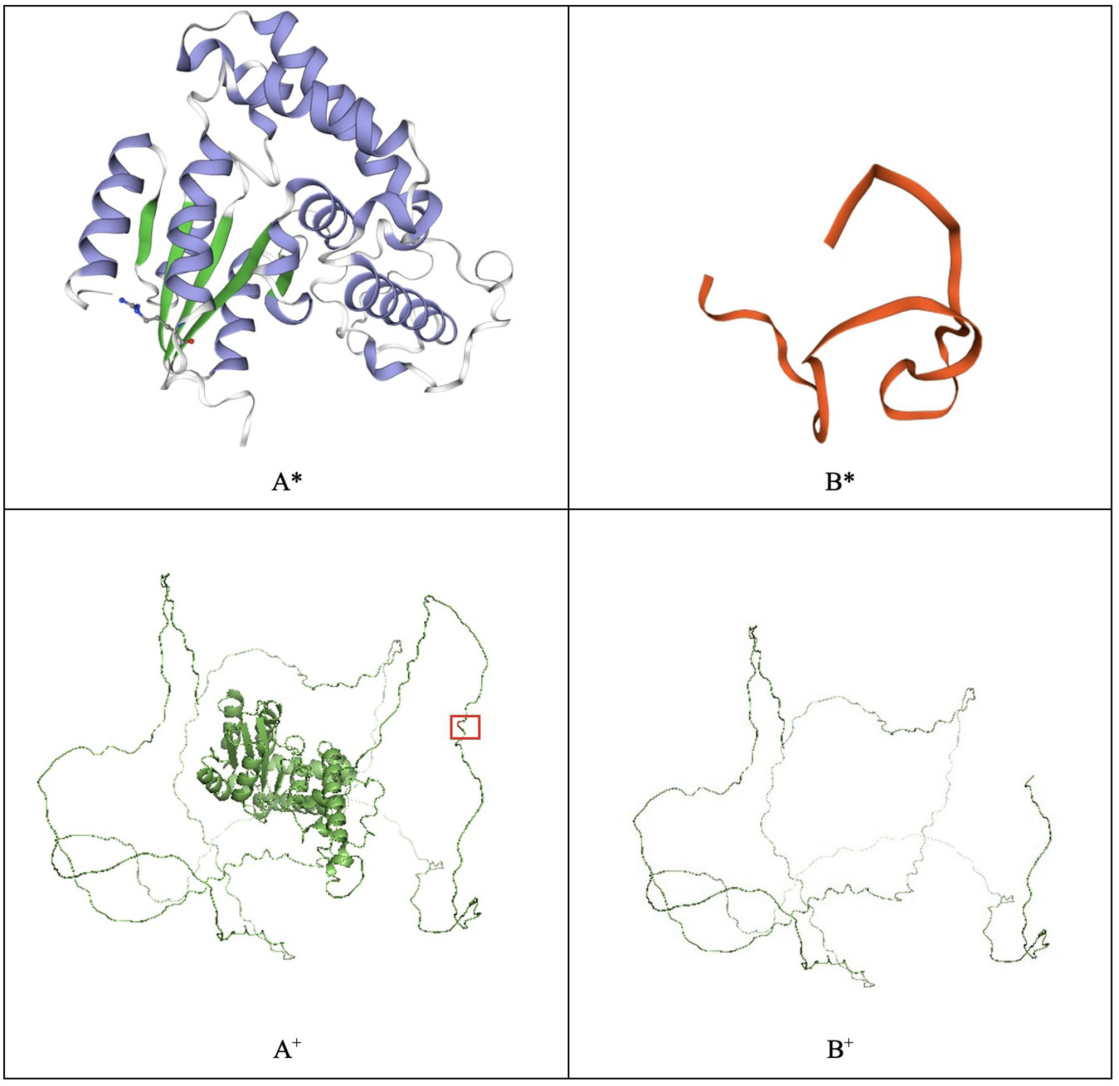
EYA4 wild-type (A) and mutated protein (B) modeled with SWISS-MODEL (*) and PyMOL (+). The mutated residue, which causes a truncated protein, is highlighted in the PyMOL wild-type model.

Several genes have been associated with hearing loss, the gene *EYA4* is one among them (4, 8). The EYA4 protein contains two critical domains. The predicted truncated protein is composed of 313 amino acids due to the mutation located on the proline–serine–threonine (PST) domain, which introduces a termination codon at residue 314 (Figure 3). *In silico* modeling was performed in SWISS-MODEL and PyMOL (9, 10). The difference between the two *in silico* approaches is that the SWISS-MODEL server uses a characterized template from its repository to model the proteins. For instance, the EYA4 protein sequence was obtained from the UniProt database entry O95677 (11) and uploaded to the SWISS-MODEL server. As a result, the server modeled the protein using the EYA2 protein as a template with a 77.62% identity, whereas the mutant protein was modeled using the TFIIIB transcription factor as a template with a 15.63% identity (9, 12), highlighting how different the mutant protein is from the wild type. On the contrary, PyMOL modeled the protein based on structure from the AlphaFold database retrieved from UniProt (13, 14). The structures depicted in thin lines have low per-residue confidence scores. The mutant residue is highlighted.

The variant has not been previously described. The Variant Interpreter platform (15, 16) predicts a likely pathogenic effect, suggesting that the lack of active and binding sites located at the eyaHR domain impedes the protein from performing any activity (17). Variants in *EYA4* have been correlated with dilated cardiomyopathy and autosomal-dominant 10 deafness (8). Autosomal-dominant 10 deafness is a form of non-syndromic hearing loss that starts in the third or fourth decade of life and is caused by a malfunction of the inner structures of the ear (8), which is consistent with the phenotype of the described subjects.

The mutation identified at the EMD protein at residue 183 does not affect the LEM domain of the protein. *In silico* modeling of the wild-type EMD protein and the mutated protein (Pro183Arg) was performed with Phyre2, SWISS-MODEL, and Hope tools. The wild-type and mutated protein models yielded similar results (9, 12, 18). Hope tool analyzed the UniProt database annotations and Reprof software predictions for mutational analysis. It determined that the mutant residue has different physicochemical characteristics than the wild type, including its size, charge, and hydrophobicity (18).

Ellis et al. (19) established that the proline residue of amino acid 183 (Pro183) is critical for the function of emerin. The researchers studied two patients with a milder form of Emery–Dreifuss muscular dystrophy and determined that both have substitutions at the Pro183 residue for either histidine or threonine (19). Similarly, Yates et al. sequenced the *EMD* gene in 22 families diagnosed with Emery–Dreifuss muscular dystrophy and found that the *EMD* gene was mutated in 21 of these families (20). However, none of these mutations in the Pro183 residue were detected or even in adjacent intervening sequences of the *EMD* gene, suggesting that this residue has functional significance for the protein. Moreover, both research groups sequenced 160 samples of people with no muscular dystrophy and did not detect the Pro183 residue.

The neuromuscular features of Emery–Dreifuss muscular dystrophy usually develop in childhood or adolescence (20). However, patients with mutations at the Pro183 residue have a late onset of symptoms (19), consistent with the appearance of the first symptoms in our subjects, who were diagnosed with muscular dystrophy at 35 and 23. The samples from subjects A and B were sequenced using the TruSight Cardio and TruSight Inherited disease kits. Both kits include genes associated with different types of muscular dystrophies (Supplementary Table 1); however, no other pathogenic mutations were found.

Ellis et al. (19) demonstrated that mutations in the *EMD* gene involving alterations in the Pro183 residue do not alter the cellular concentration or the size of the mutant emerin. However, alterations in this residue disrupt the strength of the emerin interaction with nuclear lamina components, alter the nuclear localization of emerin, and cause the mutant protein to be more hydrophilic than the wild type (19).

Emerin is a type II integral membrane protein part of the inner nuclear membrane. Neither of the two subjects studied by Ellis et al. presents a reduction in emerin expression levels, or retention of emerin in the endoplasmic reticulum, suggesting that the two mutant forms of this protein are not toxic or misfolded (19). Moreover, the membrane-binding signal (27 last amino acids of emerin) has no alterations, suggesting that the emerin protein is mislocalized in these subjects, weakening the binding interactions necessary to retain emerin in the inner nuclear membrane. The Pro183 residue is located in the large hydrophilic domain of emerin, and mutations in it probably interfere with its interactions with other proteins, causing mislocalized cellular distribution.

The subjects studied by our research group presented a mutation in the Pro183 residue; however, the alterations were not for histidine or threonine, which have been previously described, but for arginine. Arginine has the same charge as histidine, although arginine is larger than histidine. Hence, it can maintain or increase the interactions with other amino acids. We suggest that the Pro183Arg mutation in *EMD* will affect protein interactions similar to those generated by the Pro183His mutation.

Interestingly, although the *EYA4* and *EMD* genes do not directly interact with each other in biological processes, mutations in both have been associated with heart defects. Furthermore, the cardiac symptoms described in the present report may be due to synergistic or overlapping effects caused by the mutations in both genes (21).

Next-generation sequencing offers several advantages over Sanger sequencing. For instance, in Sanger sequencing, information regarding the gene and the variant position is required, whereas, in NGS, due to its high yield, it is possible to analyze the entire genome, looking for variants that may be associated with the subject's phenotype (22). However, NGS has limitations, such as the need for bioinformatics tools to process the data generated or the presence of incidental findings or variants of uncertain significance (22, 23).

Moreover, ancestry analyses were performed, and Subjects A and B present a major African component with 46 and 41.3%, respectively. According to the Pan American Health Organization (PAHO), Sub-Saharan Africa reports the highest incidence of hearing loss in the world, along with South and Pacific Asia. However, the principal cause of deafness in the region is untreated ear infections (24). On the contrary, Latin America and the Caribbean account for only 9% of the global burden. Further studies should be performed to understand the correlation between ancestry and hearing loss. Additionally, Oliveira et al. reported an important lack of epidemiological data, which complicates the understanding of diseases as EDMD and the association between the disease and the ancestral component (25).

## Conclusion

The present case report describes two unreported variants in Ecuadorian siblings presenting similar symptoms. The first mutation (NM_001301013.1:c.940G>T) in the gene *EYA4* causes a truncated protein, which could be associated with the deafness phenotype in both subjects. Moreover, the mutation (NM_000117.2.c.548C>G) in the gene *EMD* suggests a correlation between the identified variant and the Emery–Dreifuss muscular dystrophy in the participants. It is fundamental to highlight the importance of genomic analyses because they could help to understand the diseases' etiology and guide physicians in the treatment.

## Data availability statement

The genomic data are available in NCBI Sequence Read Archive (SRA) with the BioProject accession number PRJNA953961. (https://www.ncbi.nlm.nih.gov/bioproject/PRJNA953961/). For more information, please contact the corresponding author AKZ anazambrano17@hotmail.com).

## Ethics statement

The studies involving human participants were reviewed and approved by the Human Research Ethics Committee CEISH (by its acronym in Spanish) of Universidad UTE (CEISH-2021-015) and (CEISH-2021-016). The patients/participants provided their written informed consent to participate in this study. Written informed consent was obtained from the participant/patient(s) for the publication of this case report.

## Author contributions

ND, AAI-R, and AKZ: conceptualization. RI-C, JLL-B, and RH: resources. AKZ, VAR-P, SC-U, PG-R, EP-C, and RT-T: methodology. SC-U and PG-R: formal analysis and writing—review and editing. AKZ: supervision, project administration, and funding acquisition. All authors read and approved the final manuscript.
