# Supplementary material for: Identification of mutations on the EMD and EYA4 genes associated with Emery–Dreifuss muscular dystrophy and deafness: a case report
Source: Front Neurol. 2023 May 12;14:1183147. doi: 10.3389/fneur.2023.1183147 (PMC10213617; doi:10.3389/fneur.2023.1183147)
Supplement: Supplementary file 1 [file Table_1.DOCX]

**Supplementary Table 1.** Sequenced genes associated with muscular dystrophies.

| **Gene** | **Type of muscular dystrophy (MD)** |
| --- | --- |
| EMD | Emery-Dreifuss MD |
| DMD | Duchenne’s MD, Becker MD |
| LMNA | Limb-girdle MD |
| CAV3 |  |
| SGCG |  |
| SGCB |  |
| SGCD |  |
| TCAP |  |
| FKRP |  |
| TTN | Tibial MD, Limb-girdle MD. |
| DES | Myofibrillary/desmin-related myopathy |
| LAMA2 | Congenital MD type 1A |
| FKTN | Fukuyama congenital MD |
| COL6A1 | Bethlem myopathy |
